# Screening single-cell trajectories via continuity assessments for cell transition potential

**DOI:** 10.1093/bib/bbad356

**Published:** 2023-10-20

**Authors:** Zihan Zheng, Ling Chang, Yinong Li, Kun Liu, Jie Mu, Song Zhang, Jingyi Li, Yuzhang Wu, Liyun Zou, Qingshan Ni, Ying Wan

**Affiliations:** Institute of Immunology PLA, Army Medical University, Chongqing, China; Biomedical Analysis Center, Army Medical University, Chongqing, China; Department of Autoimmune Disease, Chongqing International Institute for Immunology, Chongqing, Chongqing, China; Institute of Immunology PLA, Army Medical University, Chongqing, China; Biomedical Analysis Center, Army Medical University, Chongqing, China; Biomedical Analysis Center, Army Medical University, Chongqing, China; School of Big Data and Software Engineering, Chongqing University, Chongqing, China; Biomedical Analysis Center, Army Medical University, Chongqing, China; College of Life Sciences, Institute for Immunology, Nankai University, Tianjin, China; Department of Autoimmune Disease, Chongqing International Institute for Immunology, Chongqing, Chongqing, China; Department of Rheumatology and Immunology, First Affiliated Hospital of Army Medical University, Chongqing, China; Institute of Immunology PLA, Army Medical University, Chongqing, China; Institute of Immunology PLA, Army Medical University, Chongqing, China; Biomedical Analysis Center, Army Medical University, Chongqing, China; Biomedical Analysis Center, Army Medical University, Chongqing, China; School of Big Data and Software Engineering, Chongqing University, Chongqing, China

**Keywords:** single-cell RNA sequencing, trajectory analysis, T cell memory, arthritis, acute kidney injury

## Abstract

Advances in single-cell sequencing and data analysis have made it possible to infer biological trajectories spanning heterogeneous cell populations based on transcriptome variation. These trajectories yield a wealth of novel insights into dynamic processes such as development and differentiation. However, trajectory analysis relies on an assumption of trajectory continuity, and experimental limitations preclude some real-world scenarios from meeting this condition. The current lack of assessment metrics makes it difficult to ascertain if/when a given trajectory deviates from continuity, and what impact such a divergence would have on inference accuracy is unclear. By analyzing simulated breaks introduced into in silico and real single-cell data, we found that discontinuity caused precipitous drops in the accuracy of trajectory inference. We then generate a simple scoring algorithm for assessing trajectory continuity, and found that continuity assessments in real-world cases of intestinal stem cell development and CD8 + T cells differentiation efficiently identifies trajectories consistent with empirical knowledge. This assessment approach can also be used in cases where a priori knowledge is lacking to screen a pool of inferred lineages for their adherence to presumed continuity, and serve as a means for weighing higher likelihood trajectories for validation via empirical studies, as exemplified by our case studies in psoriatic arthritis and acute kidney injury. This tool is freely available through github at qingshanni/scEGRET.

## INTRODUCTION

Since its advent, single-cell RNA sequencing (scRNAseq) has been applied across a wide range of contexts to map the cellular diversity of biological systems under physiological and pathological conditions [1–3]. Beyond atlas studies, however, the development of trajectory inference (TI) algorithms has made it possible to arrange scRNAseq cell populations into ordered hierarchies [4, 5]. These TI algorithms can model trajectories of cellular development and/or differentiation, and identify possible transitions between cell states. Trajectory analysis has already been applied in diverse biological contexts to interrogate longstanding questions of lineage origin. For instance, trajectory analysis of embryonic development has been used to resolve the initial differentiation of diverse stromal progenitor cells contributing to tissue specification [6, 7]. Lineage tracing in hematopoiesis has answered a longstanding question regarding myeloid lineage differentiation [8, 9] and clarified the mechanisms enabling turnover of tissue-resident macrophages [10, 11]. Mapping of T cell trajectories in tumor microenvironments has identified key mechanisms driving CD8 + T cell exhaustion [12, 13]. The promising insights gained from these and other studies have encouraged the application of trajectory analysis as a significant component in a large portion of scRNAseq studies.

To optimize the efficacy of trajectory analysis and improve identification of trajectories corresponding to biological reality, benchmarking studies have previously emphasized the importance of algorithm selection to fit topologies [14]. For instance, while some TI tools are designed to identify tree-like structures, others may be better suited for contexts involving cycling paths and/or other complex tree-independent topologies [15]. Other authors have developed tools for batch effect correction that filter out unwanted source of variation in larger-scale single-cell experiments to preclude trajectories distortion by technical noise [16]. However, the influence of other underlying factors on the accuracy of trajectory analysis is incompletely understood. In particular, most TI algorithms have shared assumptions of homoscedasticity and continuity along a given trajectory, which requires all intermediate states between a given start and end state to be sampled. Generally, methods that align trajectories through principal curves presume homoscedasticity across all points on the curve, and missing an intermediary cell state would introduce at least one point of excess variation. Similarly, measures of distance between pairs of points that graph- and tree- based approaches rely on would be distorted by any missing populations. Unfortunately, the true impact of discontinuity on the accuracy of trajectory analysis and inferences of cell state transitions has not yet been assessed in detail.

The uncertainty surrounding the influence of continuity on TI algorithms is particularly problematic given that real world data are susceptible to sampling limitations that may contribute to disjointed capture of cell states. In particular, while the objective of scRNAseq experiments may be to ideally census all relevant cell states, not all transitions necessarily occur within a single same site or timepoint. For instance, maturation of some cells is known to require migration from niches in one tissue to another, such that a single-tissue sample would naturally fail to capture key intermediates [17]. Other technical limitations, such as population rarity, efficiency of lineage tracing and varying sensitivity to processing, may also contribute to the unperceived loss of some populations. More vexingly, while omission of an intermediate cell type might be noticeable based on prior knowledge in well-studied systems, the lack of independent assessment metrics makes it impossible to ascertain if a given trajectory is in fact continuous when analyzing the unknown. This deficiency makes it difficult at present to discern the optimal trajectory(es) for downstream validation work and has resulted in a number of fruitless chases [18]. This limitation thus significantly hampers the current predictive utility of TI.

In this manuscript, we sought to explore in detail the impact that the loss of intermediate cell state(s) and deviation from homoscedasticity have on the accuracy of trajectory analyses. We then develop an analysis approach to highlight potential deviations from continuity in single-cell trajectories by calculating cumulative expression changes between pseudotime steps. We then apply this approach to screen potential trajectories in distinct biological settings to help identify those with the most potential to encompass direct cell state transitions, including the contexts of intestinal stem cell differentiation, T cell differentiation and podocyte response to injury.

## METHODS

### Synthetic scRNAseq datasets

To generate synthetic single-cell datasets for initial testing, we applied the splatter package [19] in R using the path simulation option to generate datasets where a reference step order for each cell would be available as a gold standard for comparison. As an approximation of a typical use case, we simulated a dataset of 1000 cells and 10 000 genes over 300 steps, allowing for 30% of the genes to be differentially expressed and a nonlinear probability of 0.25 for gene expression to enable sufficient noise. Three groups were simulated, with the intermediate group accounting for a smaller fraction of cells (20 versus 40% for the two ends). The intermediate group could then be withheld to evaluate the influence of discontinuity on the accuracy of TIs.

### Real-world scRNAseq datasets

For further evaluations of the impact of missing intermediate cell types on TI accuracy, we downloaded and processed several publicly available datasets for independent re-analysis. Single-cell RNAseq libraries of three murine intestine epithelial cell preparations [20] were downloaded from GEO accession GSE152325 as cellranger-processed matrix outputs. Multi-omics scRNAseq and scTCRseq of healthy human donor CD8+ T cells were downloaded from the 10X dataset resource [21]. Multi-omics scRNAseq and scTCRseq of paired synovial fluid and peripheral blood samples from human patients with psoriatic arthritis (PsA) [22] generated using 10X Chromium were downloaded from EBI accession E-MTAB-9492. scRNAseq libraries of human renal samples in patients with acute kidney injury (AKI) and controls [23] were downloaded from GSE210622. All expression matrices from each study were processed through the Seurat package [24] in R. Each dataset was then filtered based on common quality metrics (number of genes detected, mitochondrial content) to retain high-quality single-cells, and highly variable genes were used for PCA-based dimension reduction. For the T cell datasets, additional filtering was performed based on TCRseq data to retain only cells with complete TCRα and TCRβ chain sequences (full V and J chain identification, and CDR3 sequences), with cells containing multiple distinct TCRβ chains also being removed. To minimize the effects of batch/sample variation, PCA dimensions were corrected using the harmony package [25] in R prior to UMAP dimension reduction using the uwot package [26]. Manual annotation of the clustered identified in the UMAP space was performed to label individual cell types, while renal cell annotations were extracted directly from the provided manuscript metadata.

### Trajectory analysis

Expression profiles of synthetic and real-world scRNAseq datasets processed as Seurat objects were exported out for trajectory analyses using several trajectory analysis algorithms. We selected several of the most commonly methods, monocle 2 [27] (a tree-based approach), TSCAN [28] (also based on minimum-spanning trees), SCORPIUS [29] (based on principal curves) and slingshot [30] (also based on principal curves) as the trajectory analysis algorithms for these analyses. For further comparison, we also utilized VIA [31], an alternative approach relying on lazy teleporting random walks. For the majority of our analysis, we applied slingshot, as this method is readily applicable for spanning trajectories in batch-corrected dimensions (e.g. those returned from harmony) and is thus faster and less memory-intensive compared with alternative approaches that also need to generate corrected expression matrices as an intermediate step. Since our assessment metric is primarily focused on evaluating the continuity of competing lineages, we primarily utilized slingshot in our real-world analysis cases involving multiple samples. While slingshot has options for predetermined start and end clusters, we elected not to use these options to instead generate a larger pool of unsupervised lineages for analysis. However, our assessment approach can be similarly applied to trajectories calculated using other TI algorithms.

### Gene expression variance with respect to pseudotime and enthalpy metric

Despite differences in algorithm implementation, the central output of TI tools is a simple vector that orders each cell with respect to gene expression. This vector can thus be understood as a cumulative order of the changes in gene expression from cell to cell. For a trajectory to be homoscedastic, the magnitude of gene expression changes from cell to cell should be highly similar across all cells. Conversely, a sharp increase in gene expression changes between any two cells (relative to the baseline) would be indicative of a point of discontinuity in the trajectory. However, given that scRNAseq data generally include large proportions of zero values and may be susceptible to technical dropout [32], direct comparisons using individual cells would be exposed to excess noise and are computationally inefficient. To avoid this issue, we first binned cells into discrete pseudotime steps to generate an average expression profile at each step. In cases where a given bin has too few cells (<3 by default), this bin is removed from consideration, as sparse bins may be susceptible to excessive technical noise. Differences in cell count between one bin and its previous bin are visualized using a colored CellNumber scale to help highlight any bin-steps involving major variation in pseudotime distribution density. We then sought to calculate the overall sum of all changes in gene expression between one step to the next, in a manner akin to calculations of the change in enthalpy for chemical reactions, as follows:


$$\Delta{\mathrm{H}}_{Step}=\left({\sum}_{n=1}^i\left|{a}_n^{\prime }-{a}_n\right|\right)\Big/i$$

where *a* is the average log-normalized gene expression of gene *n* at the initial step, and *a’* is its expression at the next step. Since the total numbers of genes detected may vary from dataset to dataset, we then divide the summed absolute difference by the number of genes considered to return a more comparable value. This approach is, for all intents and purposes, very simplistic. However, we believe its simplicity may also be its greatest virtue, as it enables quick runtimes and a more intuitive metric.

### scEGRET package

To enable the easy implementation of our expression grading of running enthalpy in single-cell trajectories (EGRET) assessment metric, we have written an R package for processing single-cell expression profiles and inferred trajectories to calculate their continuity. A matrix of raw expression counts/TPM data, a vector of pseudotime values and optional associated metadata are collected to generate an EGRET object using the egret.flap command. All calculations can then be performed using a single command (egret.fly), including DEG testing and enthalpy calculation. Various visualizations can then be depicted using a number of ‘soar’ commands. From our testing, runtime using EGRET of a single-cell dataset of ~5000 cells and 10 000 genes post-filter can be completed in ~5 min using a personal computer, with the primary limitation being memory. Downstream visualizations are drawn using the ggplot2 [33] package in R, with requirements for some extra plug-ins such as ggstream [34] and ggpubr [35]. These include pyramid plots that can be used to visualize the change in enthalpy, as well as other metrics of gene expression similarity, across pseudotime bins.

EGRET also contains several convenience functions to identify genes with expression changes correlated with trajectory progression (FindCorrelatedGenes), and genes that show steady expression across the entire trajectory (FindBackgroundGenes). The former is similar to the feature to find trajectory-associated genes common to many TI packages, while the latter is helpful for identifying reference background genes for validation work (e.g. selection of context-optimized housekeeping genes for qPCR or background correction for enrichment analyses). Streamplots capturing the general trend of changes between cell populations along a trajectory as an esthetically intuitive visualization. The package and underlying source code is available through github at qingshanni/scEGRET. We welcome any interested users to contact us should any issues arise.

## RESULTS

### Trajectory continuity is required for accurate TI

**Figure 1:**
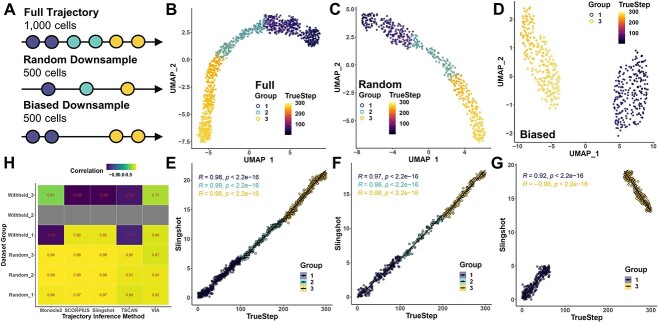
Missing intermediate cell states disrupt the accuracy of single-cell TI. (**A**) Schematic of the simulated datasets generated for assessing the influence of a lost intermediate state on the accuracy of TI algorithms. An initial dataset of 1000 cells was generated along a defined path of 300 steps in splatter to span 3 consecutive cell states (full). This dataset was then randomly downsampled down to 500 cells irrespective of cell state (random), or otherwise downsampled to include only the 500 cells at both ends of the trajectory (biased). (**B–D**) Visualizations of each of the three datasets in UMAP space following dimension reduction. Fill color of each cell corresponds to its true simulated path step, while border color and shape corresponds to its cell state. When the full dataset is analyzed (**B**), a relatively clearly defined arc can be observed. This shape is generally preserved following random downsampling (**C**), but is lost when the intermediate state is excluded (**D**). (**E–G**) Correlation analysis of the pseudotime ordering of each dataset with their true simulated path step, with each cell annotated by group. While pseudotime inferences are tightly aligned with real trajectories in the full (**E**) and random (**F**) datasets, this alignment breaks apart in the biased (**G**) dataset such that the end steps become highly negatively correlated instead. (**H**) Heatmap of the correlations between pseudotime and true simulated path step for each cell state calculated using five distinct TI algorithms.

As an initial exploration of the impact that a missing intermediate cell state may have on the accuracy of trajectory analysis, we first generated a synthetic dataset of 1000 cells spread across three states, and then downsampled this dataset in random or step-biased manners (excluding the second cell state) (Figure 1A). Dimension reduction of these datasets using UMAP yielded one relatively continuous grouping of cells when randomly downsampled, but returned two separated clusters in the biased downsample (Figure 1B–D). More importantly, TI using slingshot (a principal curves-based algorithm) was able to recapitulate the true progression order of each cell state in the full and randomly downsampled datasets with high fidelity (Figure 1E–F). However, when an intermediate state was withheld, the TI algorithm became incapable of determining the true orientation of one cell state relative to the other and returned a distorted trajectory (Figure 1G). Through further evaluations, we found that this deviation was not unique to slingshot; SCORPIUS, another principal-curve based method, was similarly unable to accurately orient the initial cell state if the intermediate cell state was withheld (Figure S1A–C). Alternative methods using minimum-spanning trees, such as monocle (Figure S1D–F) and TSCAN (Figure S1G–H) also failed to accurately infer trajectories when the intermediate state was withheld (Figure 1H). VIA, an algorithm using random walks, was slightly more robust in terms of correlation, but was nonetheless distorted to a nearly linear slope for the end cell state (Figure S1J–L). Similar trajectory errors were observed when four cell states were simulated (Figure S2). Collectively, these toy simulations demonstrate that a missing cell states or breaks may indeed introduce significant deviations in trajectory analysis even in cases involving straightforward linear trajectories. While these examples are highly simplistic, they nonetheless highlight a critical requirement in single-cell trajectory analyses for continuous trajectories.

### Gene expression-based metric flags withheld cell types along trajectories

While the results above demonstrate that missing transition states may have a major impact on the accuracy of TI, it is unclear how these breaks can be detected in a real-world context, where it is typically unknown whether any given dataset is missing key intermediate cell states. As such, we next sought to establish criteria for examining single-cell trajectories that could highlight the presence of missing cell states. To do this, we first binned cells into a discrete number of pseudotime bins of even width, before computing the averaged gene expression profiles for each bin (to help overcome issues caused by technical dropout in single-cell data).

**Figure 2:**
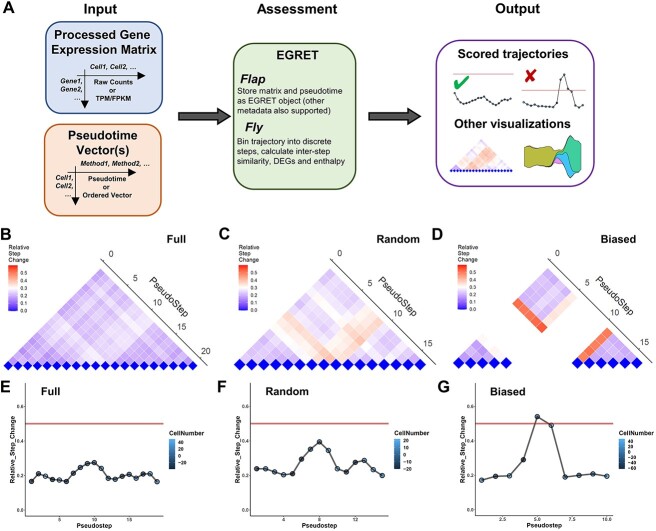
Expression-based change metric flags single-cell trajectories with potentially missing intermediate states. (**A**) Schematic of our trajectory assessment approach. By taking in a raw expression matrix containing cells of interest, and an ordered pseudotime vector for these cells, we build an EGRET object using egret.flap. Three classes of variation are calculated across each pseudotime bin using egret.fly, including the expression enthalpy metric. Downstream visualizations can be rendered using ‘soar’ commands, including line plots tracking possible breaks in continuity, as well as streamplots and pyramid heatmaps. (**B–D**) Pyramid heatmap of the relative step change values across all bins of a pseudotime trajectory calculated from the simulated datasets in Figure 1. The second-lowest level of bins in each pyramid corresponds to the overall expression difference between directly neighboring bins, while higher levels of the pyramid correspond to more distant neighbors, with the distance increasing by 1 for each level. Since the number of bins for each trajectory is computed according to the total number of cells analyzed, the full trajectory in (**A**) is calculated with 20 bins. The downsampled datasets were each calculated using 16 bins. However, some of these bins contained almost no cells in the case of the biased trajectory (**C**). (**E–G**) A simplified visualization of the step changes across directly neighboring bins in each trajectory. A cutoff flag of 0.5 is marked by the horizontal line in each of these plots as a basic reference.

We then applied different criteria, such as calculating the number of differentially expressed genes (Figure S3A–C), overall gene expression similarity (Figure S3D–F) and cumulative gene expression difference (Figure 2A–C), applied across the pseudotime bins. Of these criteria, we observed that the cumulative difference metric was sufficient to effectively mark a break point in the pseudotime trajectory that corresponded with the point at which the intermediate cell state was lost (as well as more generally flagging the existence of a break) within the outermost level of pseudotime bins (Figure 2D–F). As this metric involved the calculation of cumulative changes in gene expression between pseudotime steps, in a manner similar to reaction enthalpies, we termed our metric EGRET. Sensitivity of the EGRET metric could also be seen when additional simulated datasets were considered (Figure S4). As such, we then sought to apply this metric to analyze real scRNAseq datasets to explore its true potential.

### Trajectory assessment of intestinal stem cell differentiation finds continuous progression from ISC to enterocytes

**Figure 3:**
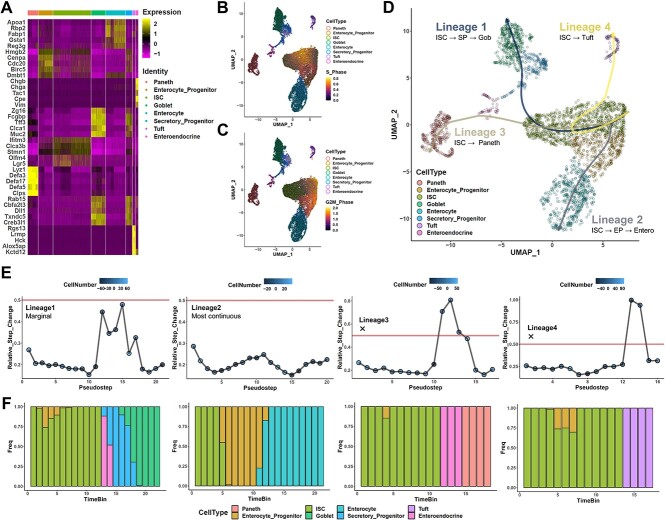
Trajectory assessment of intestinal stem cell differentiation finds continuous progression from ISC to enterocytes. (**A**) Heatmap visualization of key markers and functional molecules associated with each of the eight annotated cell types supporting their assignment. (**B–C**) Cell cycle scoring of each cell based on expression of S phase (**B**) G2-M phase (**C**) molecules overlaid in UMAP space. Values correspond to gross expression percentage of these molecules among all expressed genes in each given cell. (**D**) Visualizations of four inferred lineages obtained from slingshot calculation of the dataset, depicted in UMAP space. Lines for each lineage were obtained directly from the principal curves recovered by slingshot. (**E**) Continuity assessment of each inferred lineage using EGRET identifies lineage 2 (ISC to enterocyte progenitor to enterocyte) to be highly continuous, while sharp differences in expression can be seen in the other lineages. (**F**) Stacked bar graphs showing the cell type composition of cells in each pseudotime bin.

As an initial proof-of-principle, we first re-analyzed a scRNAseq dataset of murine intestinal epithelial cells. The small intestine epithelium is a unique, rapidly renewing biological system where a small pool of stem cells in each crypt act as multipotent progenitor for all other stromal cells in the crypt, and differentiation is widely understood to take place locally [36, 37]. These mature stromal cell types have largely been classified into secretory and absorptive lineages, and many studies have previously identified markers for each cell type involved in this system, as well factors contributing to their differentiation [38, 39]. As this dataset is composed of three independent samples and showed batch effects when uncorrected, we performed batch correction prior to analysis (Figure S5). Through dimension reduction and clustering on the corrected dimensions, we could recover clusters corresponding to the known major cell populations of the epithelial layer, including stem cells (ISC), paneth cells, goblet cells, tuft cells, enteroendocrine cells, enterocytes, as well as enterocyte progenitors and secretory progenitors (Figure 3A). Consistent with current understanding, both secretory and enterocyte progenitors were largely composed of cells with high cell cycle activity, while the mature fates were largely quiescent (Figure 3B–C). Slingshot-based TI on the UMAP projection returned four primary lineages originating from ISCs, leading to goblet, enterocyte, paneth and tuft cell fates (Figure 3D). Scoring each lineage using EGRET found that the enterocyte differentiation lineage was highly continuous, while notable breaks could be seen in the paneth and tuft cell lineages, and the goblet lineage falling in between (Figure 3E). Highly similar results could be obtained if each sample was instead considered individually, showing a robustness to our calculation using real-world scRNAseq data (Figure S6).

These results are consistent with the longstanding understanding that Paneth and tuft lineages also typically require a transition through secretory progenitors under physiological conditions [40, 41], as secretory progenitors were bypassed in these two flagged trajectories. And while the goblet trajectory did include secretory progenitors, this trajectory also included enteroendocrine cells as an intermediate state to this lineage, which is not consistent with current understanding (Figure 3F). While a study of the existing literature would have similarly identified issues with the three lineages marked as problematic from our approach, the EGRET metric raises warning flags that useful as an unsupervised indicator. As a further check that our method is indeed sensitive to missing intermediate states, we then further assessed the ISC to enterocyte lineage trajectory upon manual omission of enterocyte progenitors. Consistent with expectations, all inferred direct ISC to enterocyte trajectories were identified to have prominent spikes in EGRET when enterocyte progenitors were missing (Figure S7). Collectively, this feasibility case study demonstrates that the EGRET metric can robustly flag trajectories in real-world scRNAseq data by identifying problematic breaks from continuity.

### Trajectory assessment of T cell differentiation uncovers physiological memory phenotype transitions supported by clonal tracing

**Figure 4:**
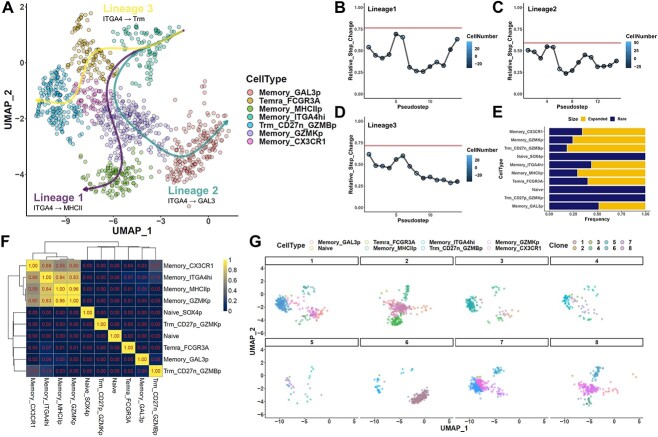
Trajectory assessment of T cell differentiation uncovers physiological memory phenotype transitions supported by clonal tracing. (**A**) Visualizations of three inferred lineages obtained from slingshot calculation of the memory/effector populations captured in the dataset, depicted in UMAP space. Lines for each lineage were obtained directly from the principal curves recovered by slingshot. (**B–D**) Continuity assessment of each inferred lineage using EGRET identifies all three lineages to be relatively continuous. (**E**) Bar graph of the proportion of cells belonging to expanded clones in each cell type. A clone (full TCRα and TCRβ match) was defined as expanded in more than three cells could be found in a single cell type. (**F**) Heatmap of clonal similarity between each cell type calculated using the Horn-Morisita index. Larger values indicate higher similarity. (**G**) Visualization of the cell type makeup of the eight largest individual clones captured in the dataset in the UMAP space of the entire dataset (Figure S8A).

While the exercise above serves as a proof-of-principle for our assessment approach, the supporting evidence is admittedly largely reliant on prior knowledge, since this system lacks a direct lineage tracer. As such, to further investigate the potential applicability of EGRET assessments, we then sought to apply our assessment to a biological system in which lineage tracing is readily available. Individual T cells are known to have their TCR sequence determined following VDJ recombination in the thymus, but may then undergo clonal proliferation and differentiation under the influence of complex factors to carry out their effector functions [42]. We thus applied our method to assess a multi-omic single-cell peripheral blood T cell dataset, in which concomitant TCR repertoire data serve as a fate tracer for individual T cell clonal lineages. Annotation of the dataset based on RNA profiles returned 10 primary cell phenotypes, corresponding to two naïve types, seven memory/effector types and an independent Trm-like cluster (Figure S8). Trajectory analysis on a random downsample of the full dataset yielded five lineages originating from naive cells, of which four involved transitions to the larger memory/effector cluster, and one a transition to the independent Trm-like cluster (Figure S9A–G). However, assessment of each lineage using EGRET returned major spikes, flagging all five lineages as likely problematic (Figure S9H–L). This result thus suggests that transition from naïve to memory/effector populations may not be favored among circulating T cells. At the same time, assessments using our approach may also have the potential to yield novel insights. For instance, if we were to perform trajectory analysis on the seven memory/effector populations that were clustered together, we could identify three lineages, all originating from the ITGA4+ cluster, that could transition into the other memory and effector lineages (Figure 4A). Scoring with EGRET showed that all three lineages passed our metric (Figure 4B–D), to suggest instead that memory populations in circulation may readily convert between each other.

To validate these inferences, we then inspected the TCR repertoires of each cell type. Through clonal analysis, we observed that the seven primary memory/effector phenotypes all included a substantial proportion of expanded clones (Figure 4E), while the naïve cluster did not. When we then performed repertoire similarity analyses between each cluster, we identified substantial repertoire overlap between each of these seven phenotypes, but almost no similarity with the naïve or isolated Trm-like cluster (Figure 4F). In particular, we could observe strikingly high clonal similarity between the ITGA4+ type and the CX3CR1+, GZMK+ and MHCII+ cell types that fall along lineage 1, but also noticeable associations along the other two lineages (while very little similarity could be found with the lineage-excluded cell types such as naïve populations). Direct inspection of the most common clones similarly supports these relations, and further suggests the possibility that some clones may simultaneously progress along one or more of the lineages (Figure 4G). This progression may be tied to the ability of ITGA4+ cells to proliferate, as cell cycle scoring of these populations showed that the ITGA4+ group had the highest proportion of cycling cells (Figure S10). Collectively, these clonal tracking concur with our trajectory-based inference that while naïve cells may not be able to readily convert into memory cells in peripheral circulation, memory populations may be able to convert amongst each other. Furthermore, individual memory cell clones may simultaneously occupy multiple phenotype states while in circulation.

### Trajectory assessment of T cell differentiation in PsA reveals patterns of clonal sharing and trajectories

**Figure 5:**
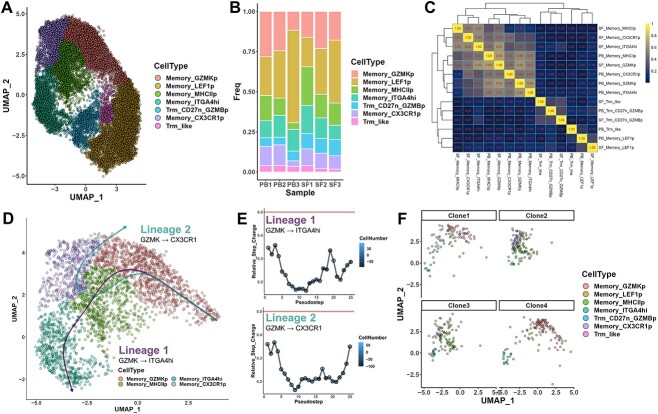
Trajectory assessment of T cell differentiation in PsA reveals lineages consistent with clonal sharing. (**A**) UMAP visualization of memory and effector CD8 + T cell types from the combined dataset of synovial fluid and peripheral blood samples of three patients with PsA. (**B**) Bar plot of the percentage composition by cell type of each sample. (**C**) TCR repertoire similarity of each cell type across the two tissues. Mean Horn-Morisita values are shown here (Figure S11B–D for individual values of each patient). (**D**) Visualizations of two inferred lineages obtained from slingshot calculation of the four cell populations showing significant clonal sharing in (**C**), depicted in UMAP space. Lines for each lineage were obtained directly from the principal curves recovered by slingshot. (**E**) Continuity assessments of the two lineages using EGRET identified both to be relatively continuous. (**F**) Visualization of the cell type makeup of the four largest individual clones captured in the dataset in UMAP space.

Since the above examples were derived from physiological conditions, we then next sought to explore the utility of EGRET for investigating pathological contexts involving T cell clonal proliferation. PsA is an autoimmune disease of the joints occurring concomitantly with dermal manifestations in a significant proportion of psoriasis patients [43]. T cells have been reported to play major roles in PsA, including tissue-resident memory CD8 + T cells [44]. An earlier scRNAseq and scTCRseq study of paired peripheral blood and synovial fluid specimens from PsA reported significant patterns of clonal sharing between specimens, but the trajectories followed by these clones have not yet been clarified. To interrogate this process, we first performed an independent re-analysis of the CD8+ T cells from the study, yielding seven population of memory cells (Figure 5A, Figure S11A), with substantial heterogeneity in these populations between samples (Figure 5B). Interestingly, through TCR analysis, we observed that four of these memory populations (MHC II+, GZMK+, CX3CR1+ and ITGA4hi) displayed high levels of repertoire similarity between each other in both peripheral blood and synovial fluid samples (Figure 5C, Figure S11B–D), indicating that many of the clones composing these populations may migrate. Trajectory analysis of these four memory populations in both tissues returned two trajectories originating from the GZMK+ population, with one leading to the ITGA4hi population (Figure 5D), and assessment using EGRET found that both inferred lineages were relatively continuous (Figure 5E), in agreement with the TCR tracking data, especially in large clones (Figure 5F). These results further demonstrate the consistency of our trajectory assessment approach with real trajectories supported by lineage tracers for investigating pathological contexts.

### Screening trajectories of podocyte response during AKI

**Figure 6:**
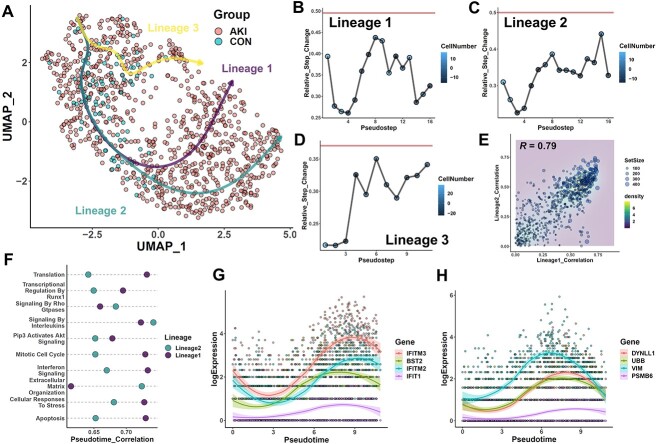
Screening trajectories of podocyte response during AKI. (**A**) Visualizations of three inferred lineages obtained from slingshot calculation of all podocytes from control and AKI patient samples, depicted in UMAP space. Lines for each lineage were obtained directly from the principal curves recovered by slingshot. (**B–D**) All three of these lineages appear to be relatively continuous when assessed via EGRET. (**E**) Pathway analysis along the two longer trajectories (lineage 1 and 2) via TIPS returned a table of correlation values between each individual annotated knowledgebase pathway from Reactome and the overall progression trajectory followed by cells in a given lineage (see Methods). Linear correlation analysis of these annotated pathways shows a strong overlap of biological processes significantly impacting the progression of both lineages. (**F**) Dotplot of some of the pathways with strongest pseudotime association with progression of both lineages. (**G**) Visualization of the expression profiles of four genes from the interferon signaling pathway over the course of pseudotime progression along lineage 1. (**H**) Visualization of the expression profiles of four genes from the apoptosis pathway over the course of pseudotime progression along lineage 1.

While the above results show that our trajectory assessment approach is useful in parsing lineages involving differentiation between distinctive cell populations, other biological questions may instead involve more transient changes experienced by a stable cell type. As an example of this, AKI, a condition characterized by a sudden loss of renal excretory function, is known to result from pathogenic insult to mature cell types in the kidneys [45]. While a large part of research in AKI has been focused on tubular cells and factors contributing to their cell death, the responses of other renal cell types are less clear. Through independent re-analysis of a scRNAseq dataset of human AKI and control kidney samples, we found that several non-tubular cells also appear to have a skewed distribution in UMAP space after correction, indicative of transcriptome variation (Figure S12A–C). This includes significant changes in podocytes (Figure S12D–G), the cells directly responsible for forming the ultrafiltration barrier in the glomerulus. While podocytes are not known to change into other cell types during AKI and are not the pathologic driver of AKI, they may potentially be involved in the post-AKI sequalae among patients with proteinuria and future renal function. Through trajectory analysis of podocyte transcriptomes, we observed three inferred lineages (Figure 6A), each of which showed continuity via EGRET (Figure 6B–D), demonstrating that our approach is also applicable in contexts involving cell state changes.

To gain additional insight into the pathways contributing to the progression of each trajectory in AKI, we then applied the TIPS package [46] to compare the influence of Reactome knowledgebase [47] pathways. Comparative analysis of the results obtained along the first two lineages demonstrated highly similar results, indicating that these two trajectories are largely governed by the same biological processes (Figure 5E). In particular, we observed significant contributions of a number of immune-related pathways along both lineages, such as interferon signaling and interleukin signaling, as well as cell death and matrix remodeling (Figure 5F). A closer inspection of the genes contributing to the interferon signaling pathway showed upregulation of several interferon-inducible factors, such as *IFITM3* and *IFIT1*, indicating the podocyte behavior may be sensitive to local interferon levels during AKI (Figure 5G). At the same time, inspection of the apoptosis pathway showed significant changes in the expression of genes associated with protein (*UBB*, *PSMB6*) and cytoskeletal maintenance (*VIM*, *DYNLL1*), with these factors peaking in expression in the middle of the trajectory. These results suggest that podocytes might undergo a choice during AKI wherein successful remodeling of intracellular components enables survival.

## DISCUSSION

Trajectory analysis has become a common component in scRNAseq analysis pipelines, and insights obtained from single-cell trajectories have helped dissect complex cell transitions. In this manuscript, we demonstrate through simulated and real scRNAseq data that the continuity of a given trajectory has a major influence on the accuracy of TI algorithms, and that the absence of continuity prevents effective inference of cellular transitions. While this result may be intuitive in the abstract, an explicit demonstration of these phenomena has, to the best of our knowledge, been lacking, especially for real-world scRNAseq datasets in which the ground truth is unknown. As such, we believe that a warning of this caveat may serve as a useful reminder regarding the need for careful implementation of analysis algorithms.

By providing an initial assessment metric to score single-cell trajectories for trajectory continuity, we make it possible to gage the likelihood of a real-world dataset to suffer from missing intermediary cell states and/or continuity defects. Our EGRET metric may be particularly helpful for parsing biological contexts in which multiple competing lineages may be inferred from a single dataset. For instance, while some TI algorithms will only return a single trajectory and pseudotime alignment for a given dataset (e.g. monocle2), other approaches (e.g. slingshot, VIA) may return large numbers of inferred lineages from a single use case. Scoring the true continuity of each lineage using our metric may thus be helpful for differentiating out lineages that meet the homoscedasticity assumption from those which fail to do so. As illustrated from our applications in intestinal stem cell differentiation and T cell activation, assessments using EGRET can help focus on trajectories supported by prior knowledge and/or lineage tracing data. Furthermore, our approach can be easily integrated into existing single-cell analysis workflows. Overall, this assessment may be helpful for comparing competing trajectories and unlock new predictive inferences in explorations of the biological unknown.

As an example of this utility, our assessments of trajectories followed by memory T cell populations in controls and PsA patients yielded some interesting insights into possible transitions between these phenotypes that are supported by TCR repertoire data. For instance, our trajectory assessments of peripheral blood and synovial fluid showed that relatively continuous transcriptome changes could demarcate the transition from ITGA4hi cells to/from those expressing GZMK, as well as cells enriched for class II MHC molecules. ITGA4 has traditionally been understood to be a marker of lymphocyte homing, as joint expression of ITGA4 and beta integrins form the α4-related integrins associated with migration to mucosal membranes [48] and other tissue sites [49]. As such, we hypothesize that circulating ITGA4hi cells may also be found in expanded numbers in the intestine. It would be interesting to explore if these clones may be cross-reactive clones that respond to both gut antigens and joint autoantigens, in which case the existence of a reservoir for these clones away from the joint may contribute to reoccurring flares of disease that persist despite therapeutic intervention. Alternatively, these trajectories may be dominated by bystander clones that are not specific for a particular local antigen, but still contribute otherwise to synovial inflammation. In such as case, the exposure of these bystander clones to inflammation in the joint may yet skew their trajectories, and cause variation in their response upon subsequent encounter with their cognate antigen. Future validation of the true extent of these transitions and the environmental milieu that may support/inhibit these changes may help to shed further insight into the true relevance of such clonal behavior in pathological contexts, particularly in terms of the intersection between infection and autoimmunity.

At the same time, we believe that it is important to emphasize that the trajectory assessment included in the present manuscript is built upon transcriptome profiles. It necessarily follows that transcriptome-based metrics like EGRET are also inherently limited to the extent that transcriptome data may be informative. However, some cell transitions may instead be more dependent upon signaling/protein -based thresholds that may not be reflected as continuous alterations in transcriptome profiles [5], and EGRET would not be particularly helpful in such cases. Due to these and other possibilities, we do not believe that it would be wise to make an absolute determination for any given inferred trajectory from transcriptome profiling alone. Instead, trajectory analysis may be more suitable as a tool for generating a pool of possible lineages, with assessment metrics serving to screen the pool for promising candidates. However, additional orthogonal indicators of cell transitions would be needed to conclusively validate a trajectory, whether flagged or not. In this regard, recent improvements in single-cell multi-omics have opened up encouraging possibilities for synthesizing in additional cell profile dimensions. For instance, as shown in our illustrations of memory T cells under physiological conditions and in PsA, concomitant lineage tracing data via TCR sequencing provided important supporting evidence for the accuracy of our assessment approach. However, one can envision that other data, such as chromatin occupancy peaks from ATACseq [50], CRISPR lineage scars recovered from genomic sequencing [51], or protein expression data, may also yield key insights. Newer analysis concepts have also made it possible more dynamic profiles by including RNA splicing and other gene regulatory activity metrics through bioinformatics [52] and labeling [53]. Finer assessments of single-cell trajectories using multiple metrics may thus vastly expand the collective understanding of developmental and differentiation processes across diverse biological settings.

**Key Points**

- Trajectory analysis based on single-cell transcriptome data has become increasingly common, but inference algorithms include underlying assumptions such as trajectory continuity that researchers need to be cognizant of
- Common TI algorithms fail when applied to disjointed trajectories in simulated and real-world single-cell data examples
- We present here a trajectory assessment metric and R package (scEGRET) that scores continuity between pseudostates along a trajectory and highlights potential discontinuity
- Continuity metrics can filter trajectories to retain optimal candidates for validation within a given dataset and are consistent with orthogonal lineage tracing data

## Supplementary Material

Suppfigures_20230727_bbad356

Supplementary_Figure_Legends_bbad356

## Data Availability

Single-cell RNA profiles analyzed in this study were accessed through public databases. Murine intestine epithelial cells were downloaded from GEO under accession GSE152325. Human renal samples were downloaded under accession GSE210622. Paired scRNAseq and scTCRseq samples from patients with psoriatic arthritis were downloaded from EBI under accession E-MTAB-9492. scRNAseq and scTCRseq of healthy human donor CD8 + T cells were directly downloaded as 10X dataset resource from the relevant application note. All other data generated in this manuscript are available on reasonable request to the corresponding authors.

## Notes

**Zihan Zheng** is a bioinformatician working on optimizing trajectory analysis methodology and using these methods to trace clonal behavior of adaptive immune cells in autoimmunity.

**Ling Chang** is a PhD student working on applying scRNAseq to interrogate T cell differentiation and identify autoreactive clones.

**Yinong Li** is a bioinformatician working on developing analysis tools for single-cell analysis and adaptive immune receptor repertoire sequencing.

**Kun Liu** is a bioinformatician working on developing analysis pipelines for single-cell multi-omics.

**Jie Mu** is a master’s student working on optimizing sequence analysis and lineage trees for adaptive immune receptor repertoire sequencing.

**Song Zhang** is a professor at Nankai University whose main research focus is on T cell biology and mechanisms driving pathogenic differentiation.

**Jingyi Li** is a professor and clinician in the Department of Rheumatology and Immunology with a research focus on identifying precision therapeutics for rheumatoid arthritis.

**Yuzhang Wu** is the director of the Immunology Institute at Army Medical University who specializes in T cell immunology during autoimmunity, chronic infection and cancer.

**Liyun Zou** is an assistant professor at Army Medical University working in the fields of antigen presentation and autoimmunity.

**Qingshan Ni** is an assistant professor at Army Medical University whose main research focus is on data analysis for high-throughput sequencing.

**Ying Wan** is the director of the Biomedical Analysis Center at Army Medical University and has led work focused on single-cell sequencing and vesicular transport in immune cells.
