# Supplementary material for: Screening single-cell trajectories via continuity assessments for cell transition potential
Source: Brief Bioinform. 2023 Oct 20;24(6):bbad356. doi: 10.1093/bib/bbad356 (PMC10589400; doi:10.1093/bib/bbad356)

FigureS1– Missing intermediate cell states disrupt the accuracy of single-cell trajectory inference independently of algorithm selection

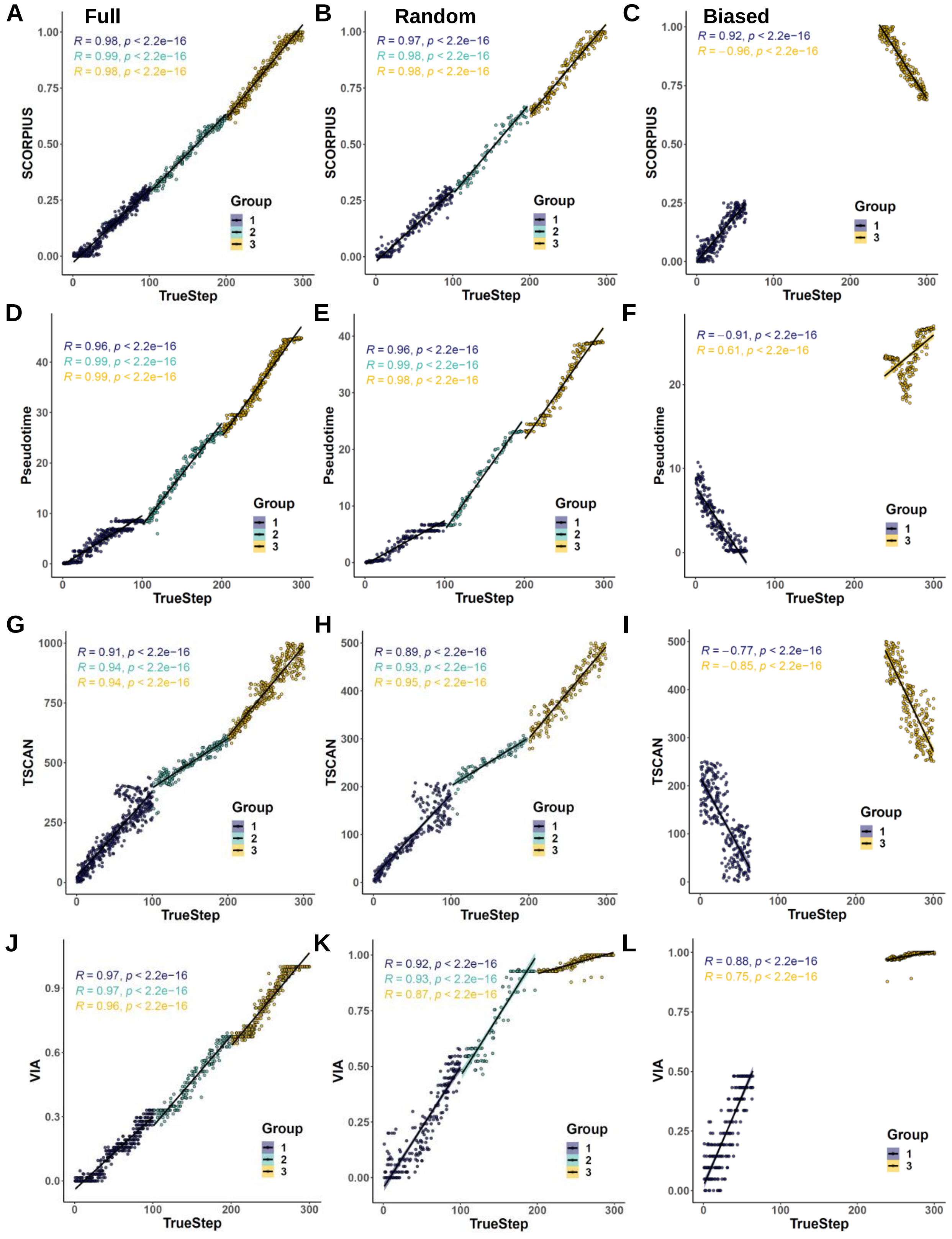

FigureS2– Trajectory distortion is evident even when breaks are weaker

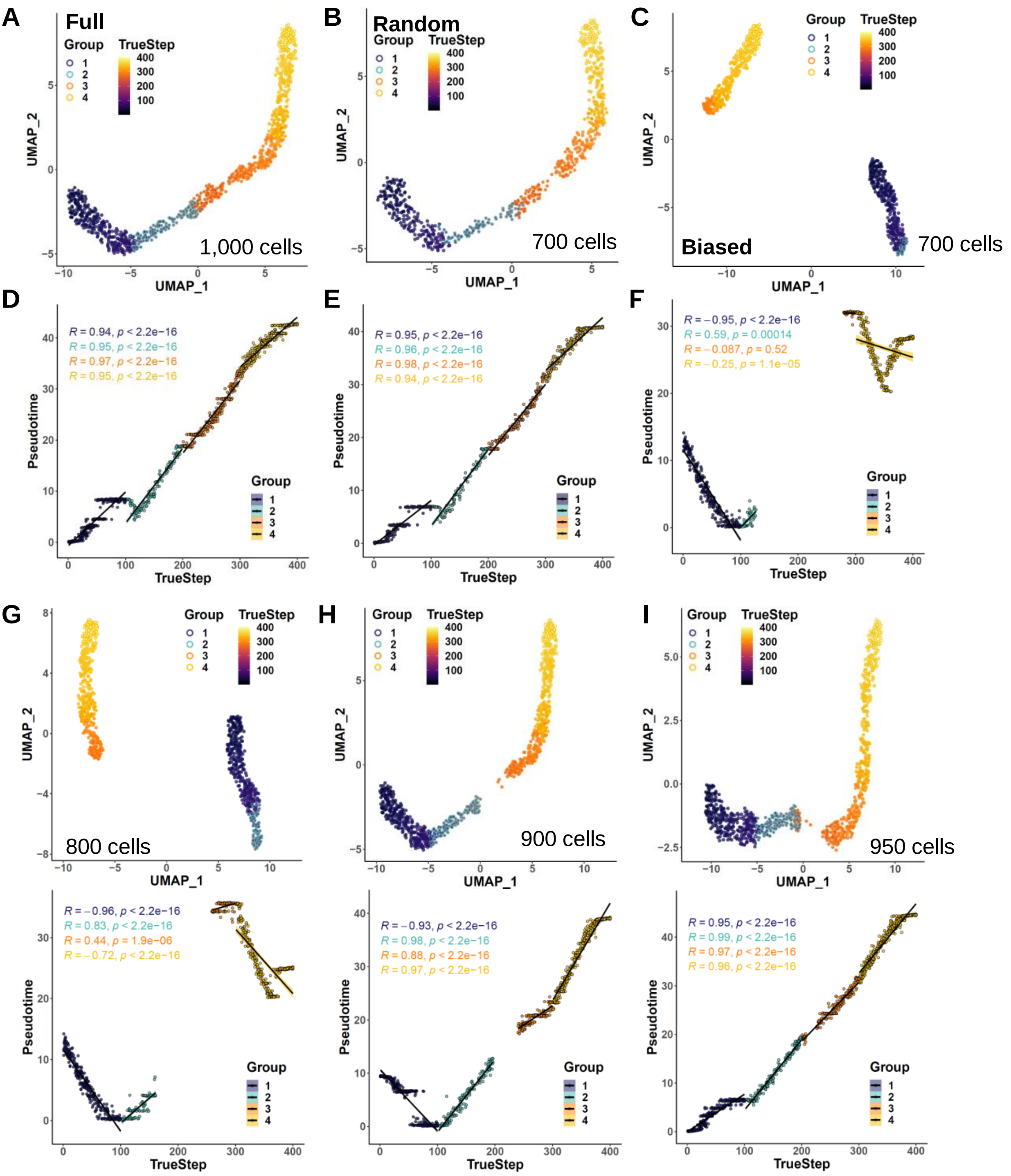

FigureS3– Transcriptome difference metric more readily finds differences in nearest pseudotime steps

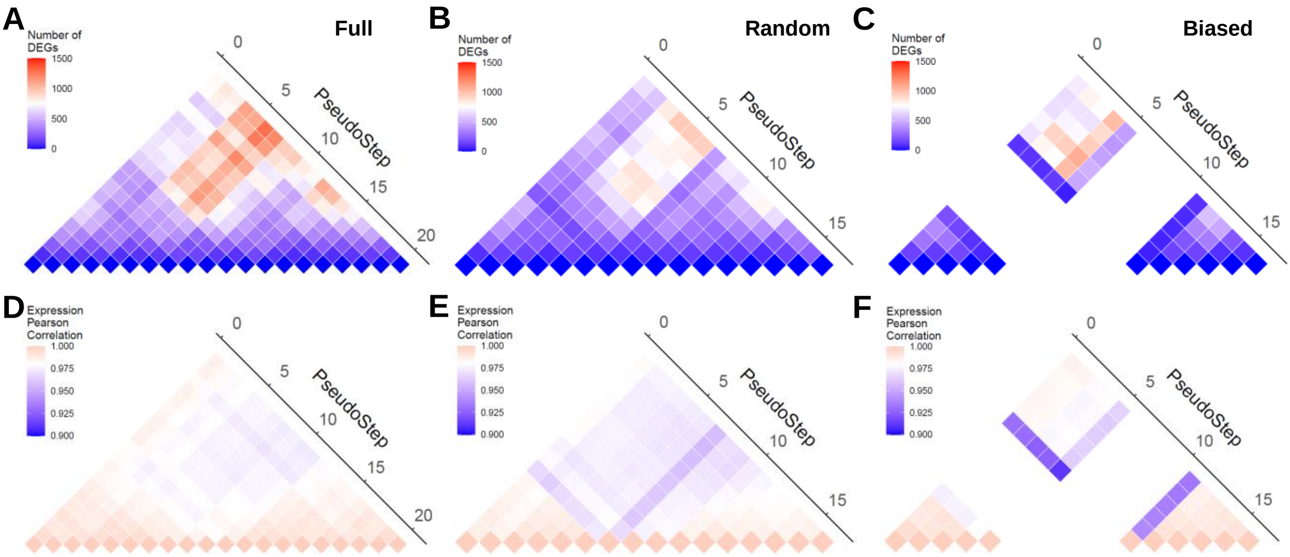

FigureS4– Transcriptome difference metric identifies break in the 4-group simulated dataset

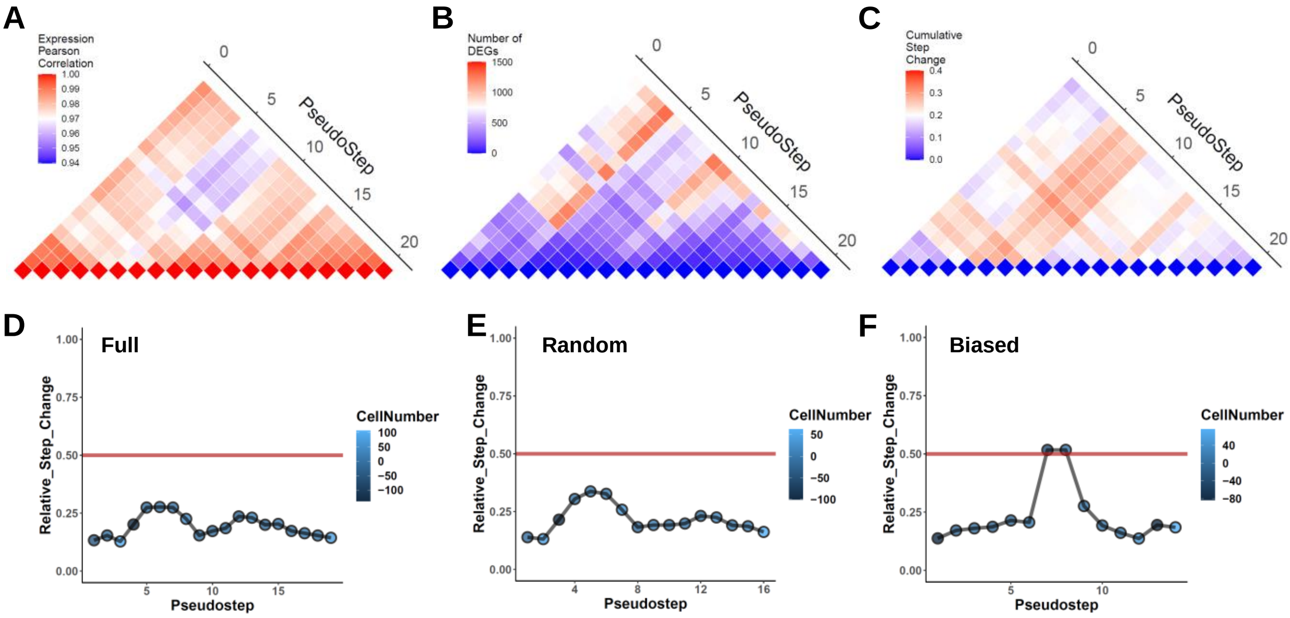

Figure S5– Batch correction for smoother trajectory analysis

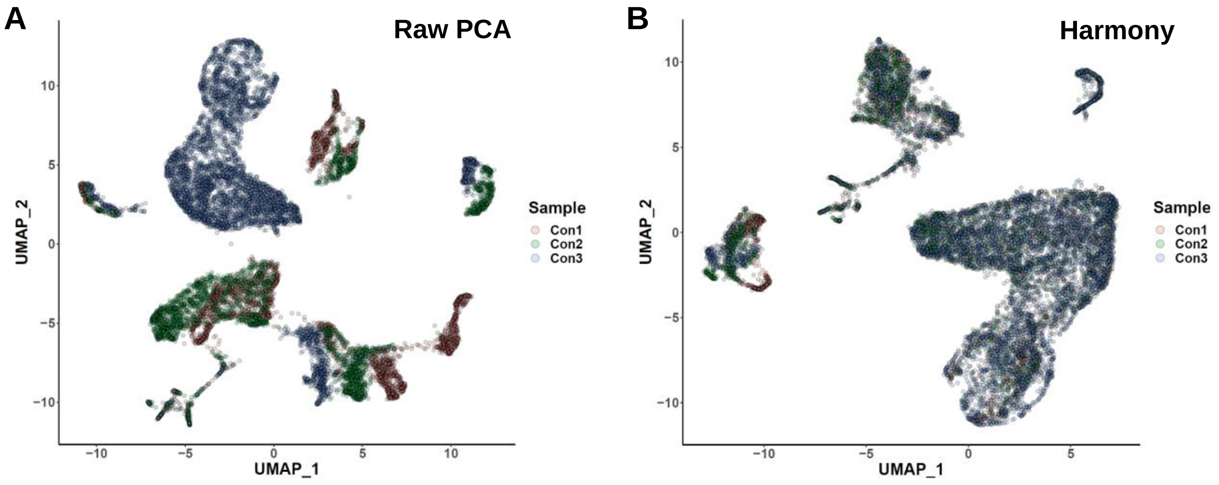

Figure S6– Individual analysis of each sample returns similar results

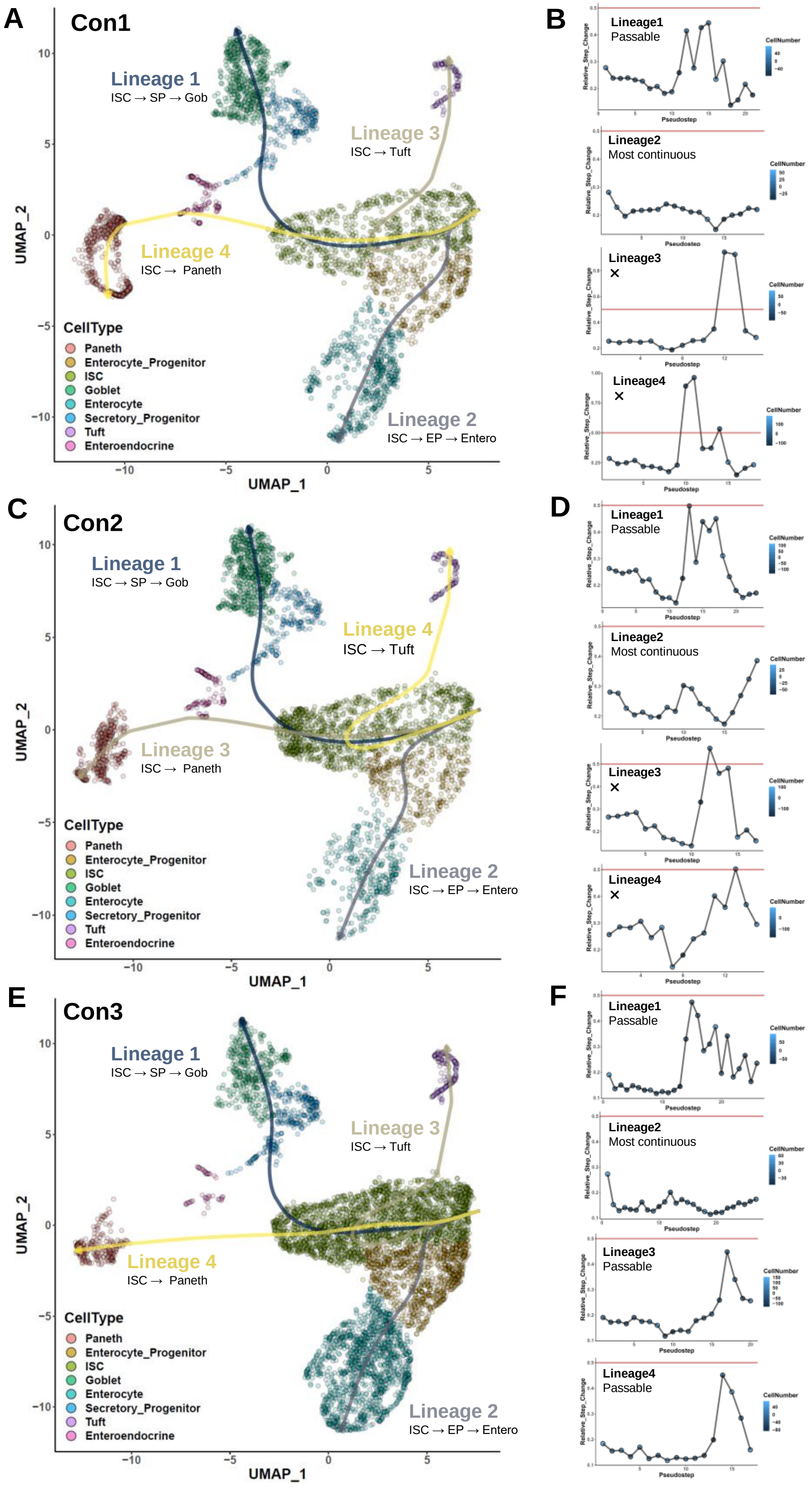

Figure S7– Withholding intermediate state causes break in trajectory towards enterocyte lineage detectable through step assessment

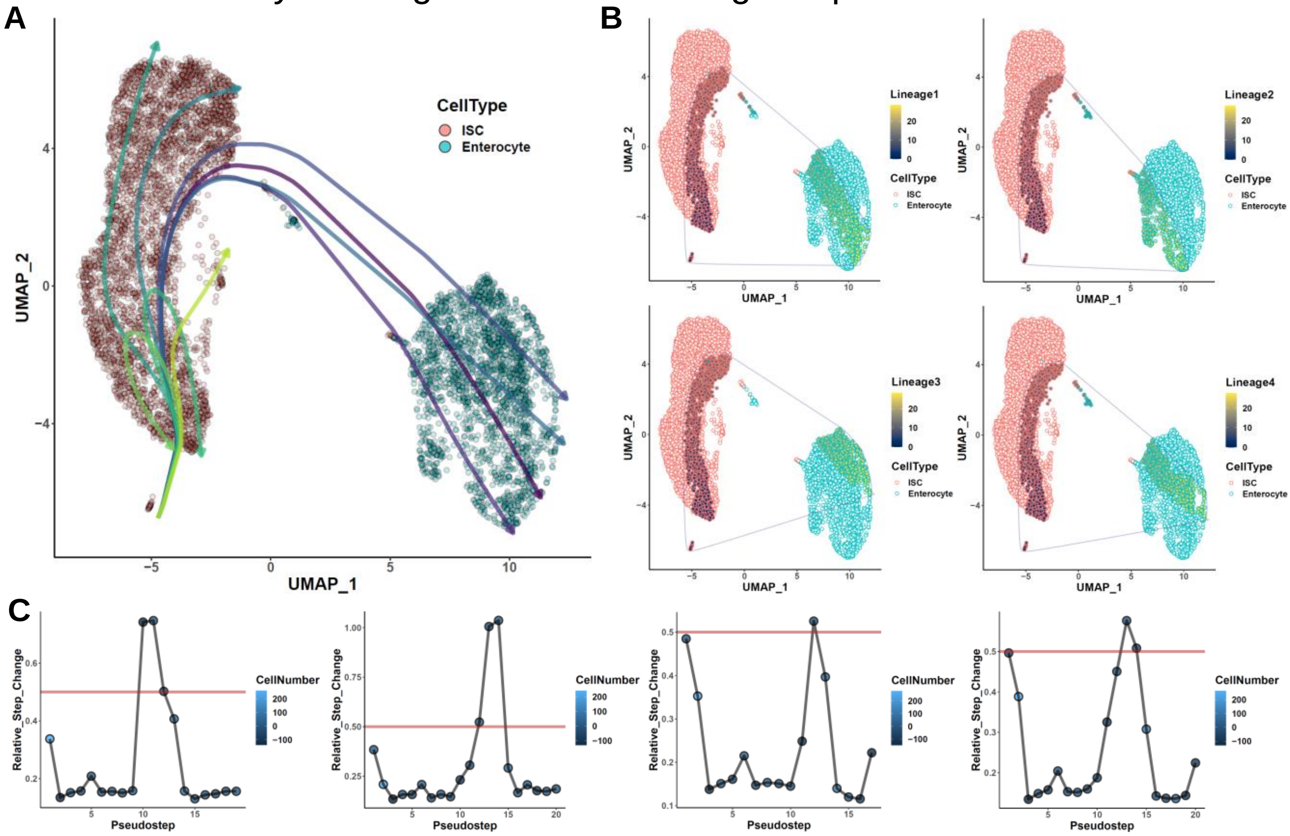

FigureS8– Annotation of CD8+T cell phenotypes

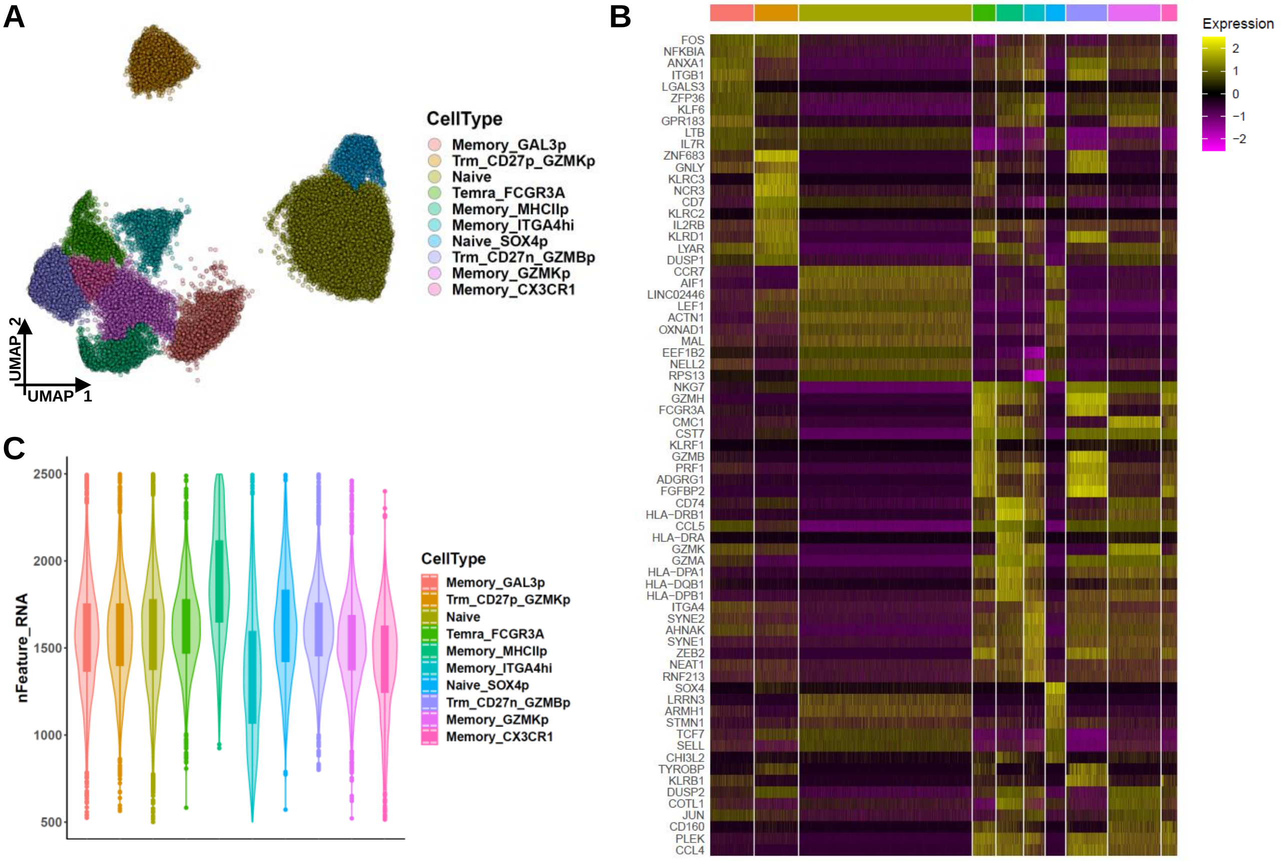

FigureS9– Slingshot elucidation of prospective differentiation trajectories among peripheral blood CD8+T cells in healthy donors

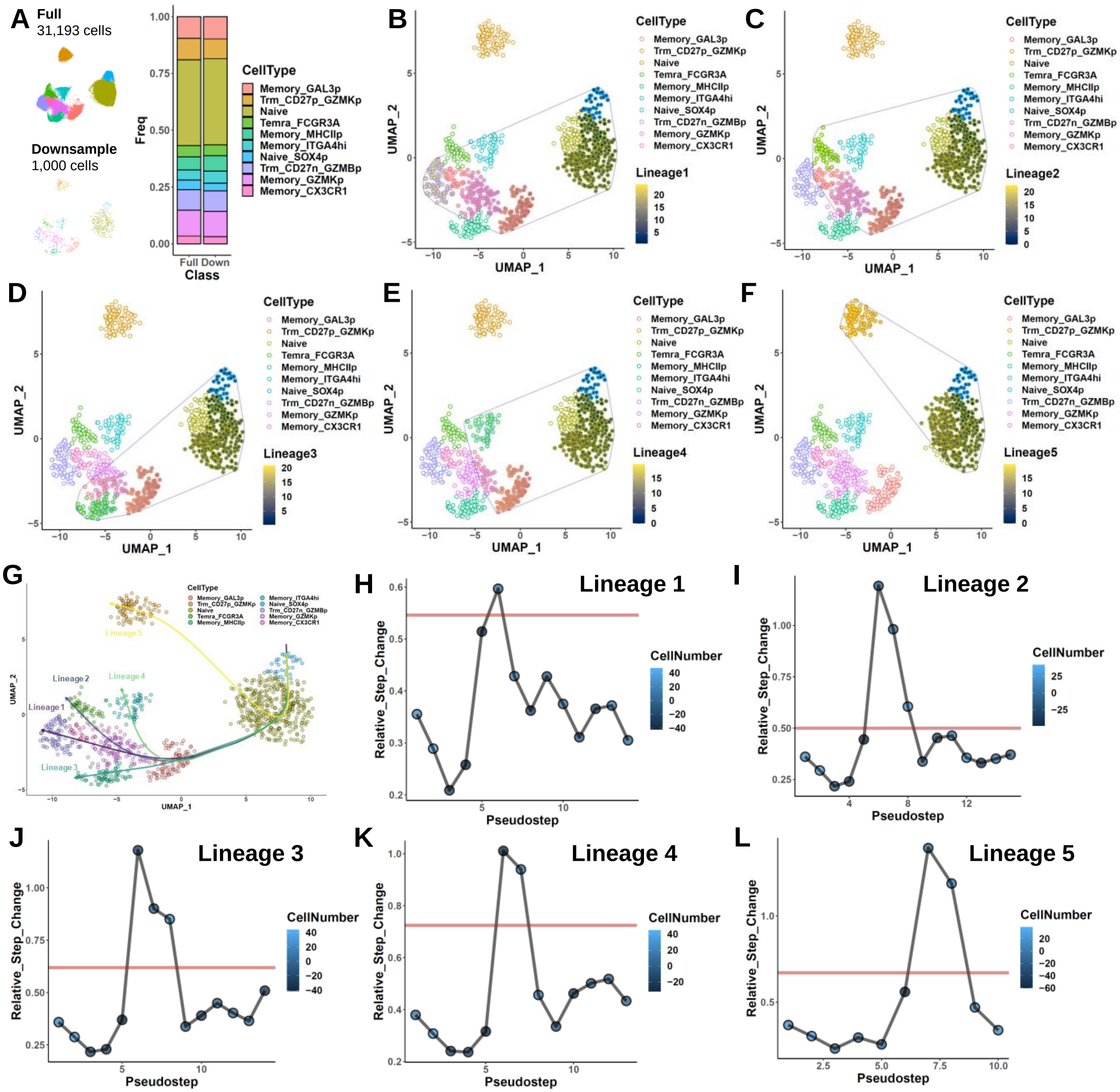

FigureS10– Cell cycle scoring of memory cells suggest potential influence of proliferation

A

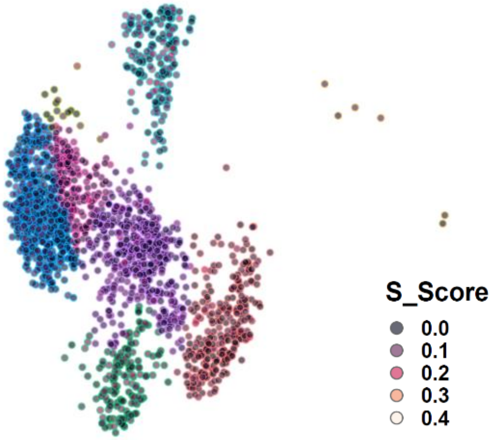

B

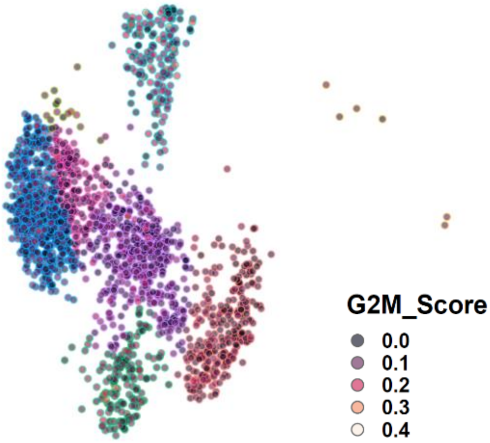

FigureS11– Sample integration and TCR clonal sharing in psoriatic arthritis

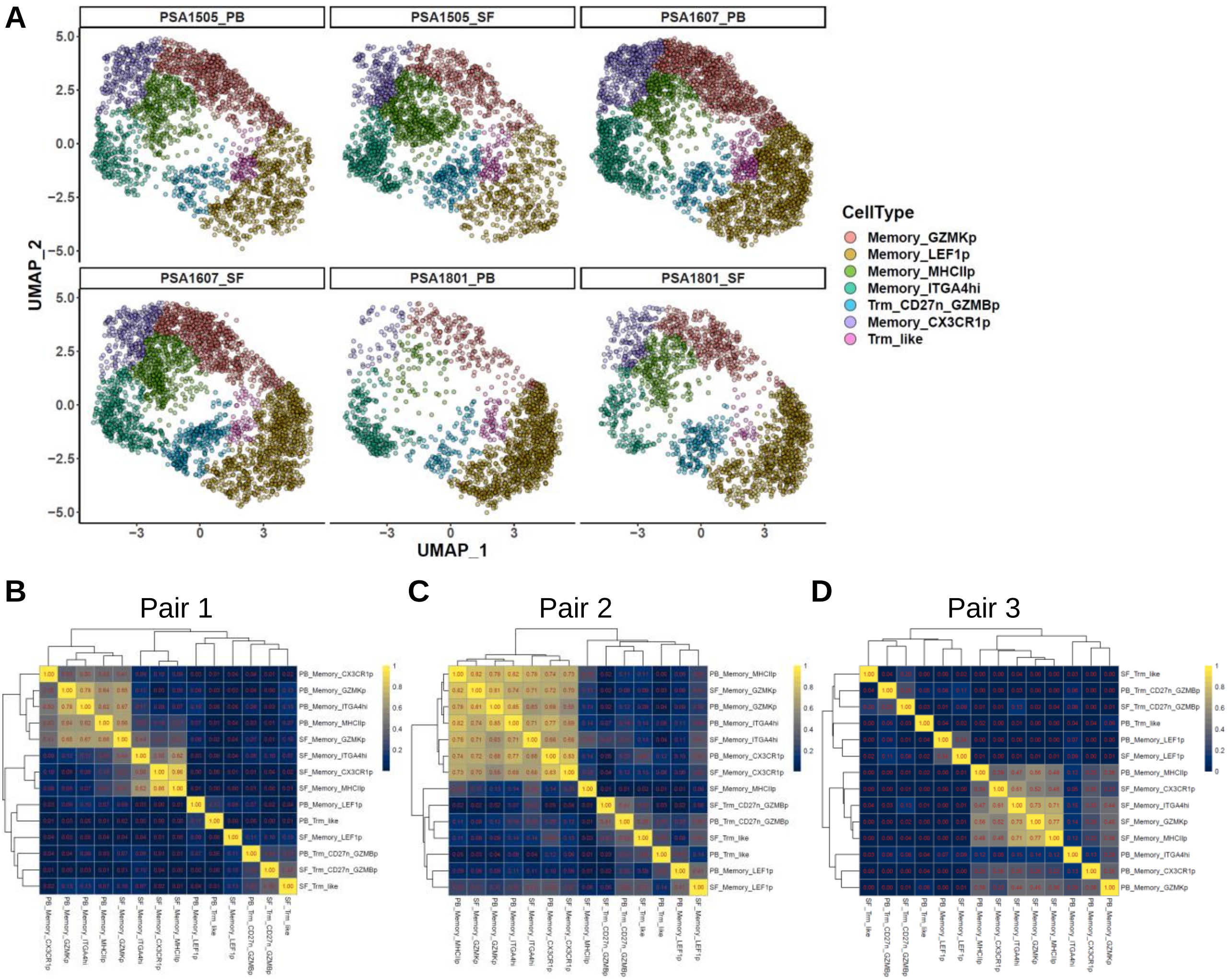

FigureS12– Trajectory analysis of human acute kidney injury

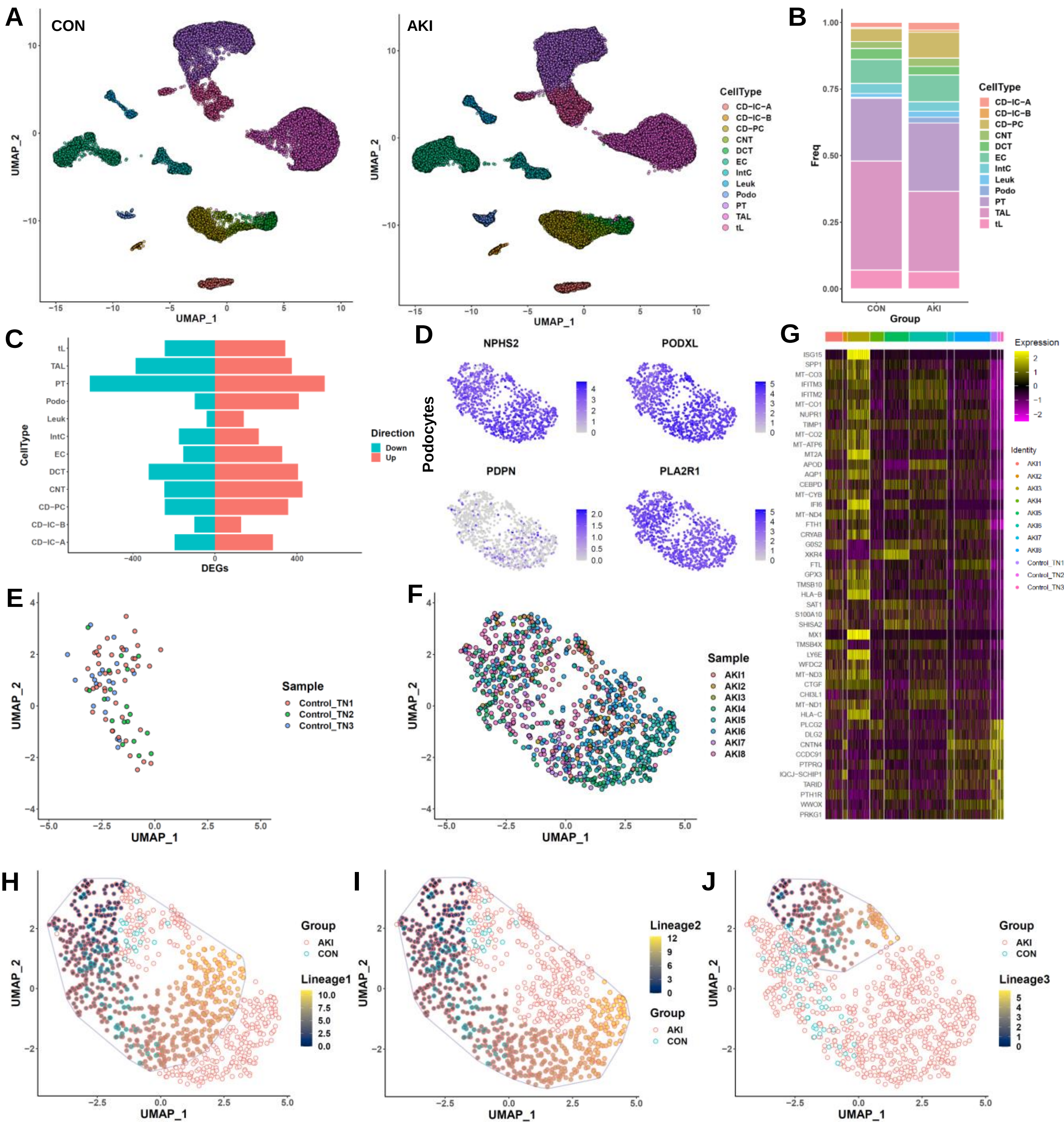

Supplement: Suppfigures_20230727_bbad356 [file suppfigures_20230727_bbad356.pdf]
