# Supplementary material for: Screening single-cell trajectories via continuity assessments for cell transition potential
Source: Brief Bioinform. 2023 Oct 20;24(6):bbad356. doi: 10.1093/bib/bbad356 (PMC10589400; doi:10.1093/bib/bbad356)
Supplement: Supplementary_Figure_Legends_bbad356 [file supplementary_figure_legends_bbad356.docx]

**Supplementary Figure Legends**

**FigureS1–** Missing intermediate cell states disrupt the accuracy of single-cell trajectory inference independently of algorithm selection

Visualization of the correlation analysis of the pseudotime ordering of each dataset with their true simulated path step based on the same dataset as Fig1 (see Fig1E-G). A-C) Correlations calculated using pseudotime ordering returned by the SCORPIUS algorithm, a principal curve-based methodology. D-F) Correlations calculated using pseudotime ordering returned by the Monocle2 algorithm, a minimum spanning tree-based methodology. Of note, additional warping of the order of group3 cells can be observed under this approach when the intermediate cell state is missing. G-I) Correlations calculated using pseudotime ordering returned by the Monocle2 algorithm, another minimum spanning tree-based methodology. Of note, pseudotime orderings in both group1 and group3 are negatively correlated with true progression. However, if we were to simply reverse the orientation of the values to return positive correlations, it would return a result that group3 cells are differentiating into group1, which would also be a nonsensical result. J-L) Correlations calculated using VIA, an alternative random walk algorithm with both lazy and teleporting steps. While the performance in group3 is improved compared to the other algorithms, the mapping in group3 is still noticeably impaired, with the slope of the linear correlation being very small.

**FigureS2–** Trajectory distortion is evident even when breaks are weaker

An initial dataset of 1,000 cells was generated along a defined path of 400 steps in splatter to span 4 consecutive cell states (full). This dataset was then randomly downsampled irrespective of cell state (random), or otherwise downsampled to include only cells at both ends of the trajectory (biased) (similar to the strategy in Fig1). By doing so, we sought to investigate to what extent a smaller gap might have on the accuracy of trajectory inference. D-F) Correlations calculated using pseudotime ordering returned by the Monocle2 algorithm still show a highly broken inference when a smaller gap of 300 cells is introduced (similar to FigS1D-F). G-I) Continued narrowing of the gap shows that a gap of 100 cells still causes improper trajectory alignment. Of note, this occurs even though all four cell states are included in the dataset (at least portions of groups 2 and 3 are included from 800 cells on up). 100 cells in this dataset essentially reflects 10% of the overall simulated dataset, indicating that even the loss of a small portion of intermediate states can still cause major issues for trajectory inference.

**FigureS3–** Transcriptome difference metric more readily finds differences in nearest pseudotime steps

A-C) Pyramid heatmap of the number of differentially expressed genes across all bins of a pseudotime trajectory calculated from the simulated datasets in Fig1 (see Fig2A-C). The second-lowest level of bins in each pyramid correspond to the overall expression difference between directly neighboring bins, while higher levels of the pyramid correspond to more distant neighbors, with the distance increasing by 1 for each level. An expression change cutoff of 0.25 and p-value cutoff of 0.05 was used. While this criteria is set to be relatively loose, more stringent fold change criteria did not lead to appreciably different results. Of note, under this criteria, the number of DEGs did not increase greatly even when crossing the introduced break in biased dataset. D-F) Pyramid heatmap as in A-C using Pearson’s correlation of mean expression profiles in each pseudotime bin as the criteria.

**FigureS4–** Transcriptome difference metric identifies break in the 4-group simulated dataset

A-C) Pyramid heatmaps calculated according to Pearson correlation (A), number of DEGs (B), and cumulative difference (C) using the four-group simulated dataset analyzed in FigS2 with a gap of 300 cells. D-F) A simplified visualization of the step changes across directly neighboring bins in each trajectory. A cutoff flag of 0.5 is marked by the red line in each of these plots as a basic reference.

**Figure S5–** Batch correction for smoother trajectory analysis

A) Visualization of the sample origin of each cell following UMAP dimension reduction of the intestine epithelium dataset using the raw PCA dimensions. Cells are highly separated based on their sample of origin. B) Visualization of the sample origin of each cell following UMAP dimension reduction of the intestine epithelium dataset following correction of the raw PCA dimensions through harmony. These embeddings return an integrated dataset that hides inter-sample variation and provide useful dimensions for meaningful trajectory analysis.

**Figure S6–** Individual analysis of each sample returns similar results

Since the trajectories analyzed in Fig3 are from the combined dataset, we also sought to evaluate if splitting it into individual datasets based on sample origin would have any impact. A,C,E) Visualizations of four inferred lineages obtained from slingshot calculation of each individual sample dataset, depicted in UMAP space. Lines for each lineage were obtained directly from the principal curves recovered by slingshot. B,D,F) Continuity assessments for each lineage for each individual sample.

**Figure S7–** Withholding intermediate state causes break in trajectory towards enterocyte lineage detectable through step assessment

A) Visualizations of the nine inferred lineages obtained from slingshot calculation of ISC and enterocytes from the combined dataset, depicted in UMAP space, recalculated following the removal of all other cell types (including enterocyte progenitors). Lines for each lineage were obtained directly from the principal curves recovered by slingshot. B) Focused visualization in UMAP space of the cells encompassed along each of the four lineages that span ISC and enterocytes, colored according to pseudotime order. C) Continuity assessments of the four trajectories shows very steep peaks with very high step change values across certain pseudotime bins.

**FigureS8–** Annotation of CD8+T cell phenotypes

A) UMAP visualizations of the CD8+T cell populations recovered from a large sequencing dataset of human peripheral blood (31,193 cells post-filter). B) Heatmap of the top markers associated with each cell type. C) Violin plot showing the distribution of number of genes detected on a per-cell basis across each cell type.

**FigureS9–** Slingshot elucidation of prospective differentiation trajectories among peripheral blood CD8+T cells in healthy donors

A) To enable more rapid calculations, we randomly downsampled the dataset down to 1,000 cells, without impacting the distribution of cells in UMAP space, or the relative proportion of cells belonging to each cell type. B-F) Focused visualization in UMAP space of the cells encompassed along each of the five lineages recovered through slingshot analysis of the downsampled dataset. Each cell is colored according to its pseudotime order. G) Overall summary of these five lineages. H-L) Continuity assessments of each of the five lineages. Each of these lineages shows sharp spikes in transcriptome difference when calculated via EGRET, with only the spike in lineage1 being relatively moderate. Of note, because the background level of variation may also vary from dataset to dataset, we took an even more flexible approach here of allowing cutoffs to rise to a step change difference value within 2 median absolute deviations of the median if such a value was greater than 0.5. However, this increased flexibility still suggested that outlier peaks were present in each of these five lineages.

**FigureS10–** Cell cycle scoring of memory cells suggest potential influence of proliferation

Cell cycle scoring of cells belonging to the eight largest clones (see Fig4G) based on built-in gene lists for S phase and G2-M phase genes from Seurat. Cells are colored based on their summed percentage expression of these genes among their total mRNA content.

**FigureS11–** Sample integration and TCR clonal sharing in psoriatic arthritis

A) UMAP visualization of memory and effector CD8+T cells in Fig5A, split into individual samples. B-D) TCR repertoire similarity calculated on the clonal level for each cell type in each pair of synovial fluid and peripheral blood samples. As each heatmap is independently clustered (per Euclidean distance), the order of cell populations varies, but a dominant cluster can still be clearly seen encompassing four cell types. PB- peripheral blood, SF- synovial fluid.

**FigureS12–** Trajectory analysis of human acute kidney injury

A) UMAP visualization of the renal dataset in patients with (n = 8) or without (n = 3) AKI. Cells were labelled according publicly available cell type annotations provided by the original authors. B) Cell type composition of all samples in the control and AKI groups. C) A general test (Wilcoxian, leveraging the built-in function in Seurat) for genes that are differentially expressed in each cell type between control and AKI patient samples (each sample considered independently). From this test, we observed that a substantial number of genes were upregulated in podocytes derived from AKI patients. D) Overlay of the gene expression of 4 markers of podocytes in UMAP space as a sanity check that these cells are in fact podocytes. E-F) UMAP visualization of the distribution of cells based on sample origin among podocytes. G) A heatmap of the top 50 markers differentially expressed in AKI podocytes compared to control. Three groups at the right are controls. H-J) Focused visualization in UMAP space of the cells encompassed along each of the three lineages recovered through slingshot analysis of the full dataset. Each cell is colored according to its pseudotime order.
